# Diagnostic and Therapeutic Challenges Associated With Left Ventricular Tumors

**DOI:** 10.7759/cureus.36443

**Published:** 2023-03-20

**Authors:** Wahab J Khan, Muhammad Asif, Ifrah Nadeem, Sadia Aslam, Mohammed A Chowdhury

**Affiliations:** 1 Internal Medicine, University of South Dakota Sanford School of Medicine, Sioux Falls, USA; 2 Internal Medicine, Avera McKennan Hospital and University Health Center, Sioux Falls, USA; 3 Cardiovascular Medicine, North Central Heart Institute, Sioux Falls, USA

**Keywords:** . radiation, cardiac papillary fibroelastoma, treatment dilemma, cardiac tumours, left ventricle mass

## Abstract

Cardiac tumors are uncommon. Sometimes it is challenging to differentiate non-invasively between different kinds of cardiac tumors and thrombi, which is critical to dictate the subsequent treatment. In addition, not all high-risk cardiac tumors are amenable to surgical resection posing a therapeutic challenge. We report a case of cardiac papillary fibroelastoma in the left ventricular cavity with a 10-year follow-up, with no embolic complications.

## Introduction

Cardiac tumors are rare, but when present, they pose a diagnostic and therapeutic challenge. Secondary or metastatic tumors are more common than primary cardiac tumors (PCT) [1]. The most common solid organ tumors to metastasize to the heart originate from the lungs, breasts, and esophagus [1]. Among the PCTs, the majority are benign [1]. The most common benign tumor of the heart is myxoma, followed by papillary fibroelastoms (PFE) and lipomas. Other less common benign tumors include rhabdomyoma and fibromas but occur more commonly in the pediatric population. The most frequently diagnosed malignant cardiac tumor is sarcoma [2]. Differentiating between these lesions and determining the most appropriate therapy could be challenging despite advances in diagnostic modalities [1]. We present such a case of a cardiac tumor with a 10-year follow-up.

## Case presentation

A 61-year-old male presented with a one-day history of worsening shortness of breath associated with weakness, fatigue, and insomnia. He had a history of chronic diastolic heart failure. A prior echocardiogram performed seven years back reported an ejection fraction of 50%-55% and a left ventricular (LV) mobile mass of 2x2 cm without regional wall motion abnormalities concerning for myxoma. He also had a coronary angiogram at that time which reported no evidence of coronary artery disease. He was previously offered surgical resection but refused further interventions. His other medical history included a prior diagnosis of Hodgkin's lymphoma 30 years ago, treated with chemotherapy and radiation complicated with radiation-induced hypothyroidism and radiation-induced lung disease, left partial pneumonectomy for blebs, T1b NX M0 renal cell carcinoma s/p left nephrectomy. He did not have a history of excessive alcohol use but had a remote smoking history. Family history was negative for any tumors or sudden cardiac death. His medication included aspirin, clopidogrel, budesonide-formoterol inhaler, carvedilol, furosemide, levothyroxine, potassium chloride, and tiotropium inhaler.

Upon presentation, Vital signs were the following; BP 100/80 mmHg, HR 124 beats/min and regular, SaO_2_ 94% on RA with RR of 27 and a T of 97.9 F. Physical examination was significant for mild distress from shortness of breath, clear lungs to auscultation and a JVD of 2 cm with minimal pedal edema. Initial labs were significant for mild leukocytosis, a new elevated hemoglobin A1c. The troponin trend was flat, and the BNP level was markedly elevated. Detailed laboratory values are given in Table 1. Initial EKG showed sinus tachycardia, RBBB, LAFB, and non-specific T-wave changes in lateral leads. Transthoracic echocardiogram (TTE) showed a 2 x 2.4 cm mobile mass in the LV attached to the anterolateral wall via a stalk without apparent myocardial invasion. LVEF was 19% with an RVSP of 46 mmHg and a mildly enlarged IVC.

**Table 1:** Laboratory values from the initial and the most recent admission. Abbreviations: WBC (white blood cells), Hb (Hemoglobin), BNP (Brain natriuretic peptide), TSH (thyroid-stimulating hormone).

Lab	Initial value	Reference value	Most recent value
TSH	2.39 uIU/mL	0.35-4.94 uIU/mL	2.57 uIU/mL
WBC	13.4 k/µL	4-11 k/µL	8.5 k/µL
Hb	14.6 g/dL	13.5-16.9 g/dL	13.8
Platelets	136 k/µL	150-400 k/µL	207 k/µL
Sodium	137 mmol/L	135 - 145 mmol/L	132 mmol/L
Potassium	4.5 mmol/L	3.5 - 5.1 mmol/L	4.6 mmol/L
Creatinine	1.4 mg/dL	0.7 - 1.3 mg/dL	1.1 mg/dL
Hemoglobin A1c	7.7%	4.0% - 5.6%	7.3%
BNP	1,167 pg/mL	<100 pg/mL	501 pg/mL
Troponin	0.33 ng/mL	<0.03 ng/mL	Not checked

**Figure 1:**
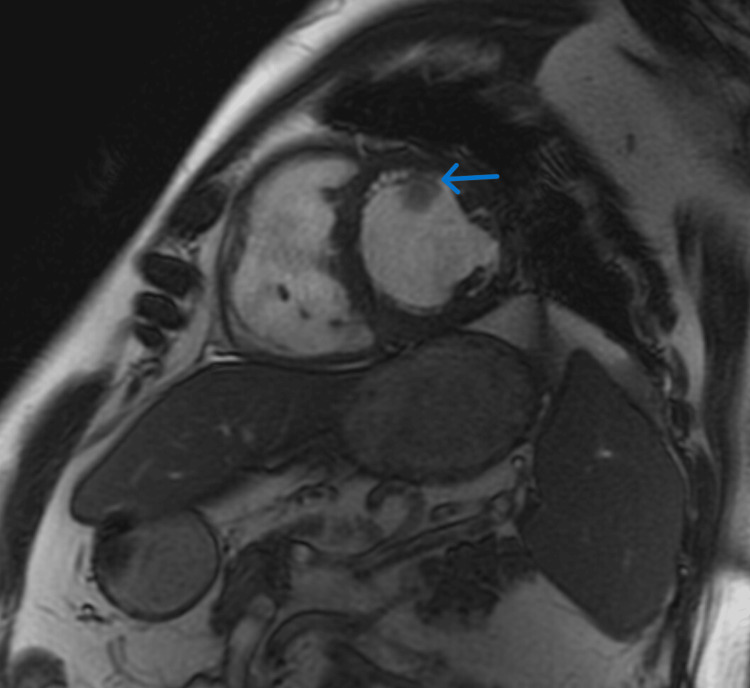
Cardiac MRI showing 17 x 17 mm mass attached to the anterior wall of the left ventricle.

**Figure 2:**
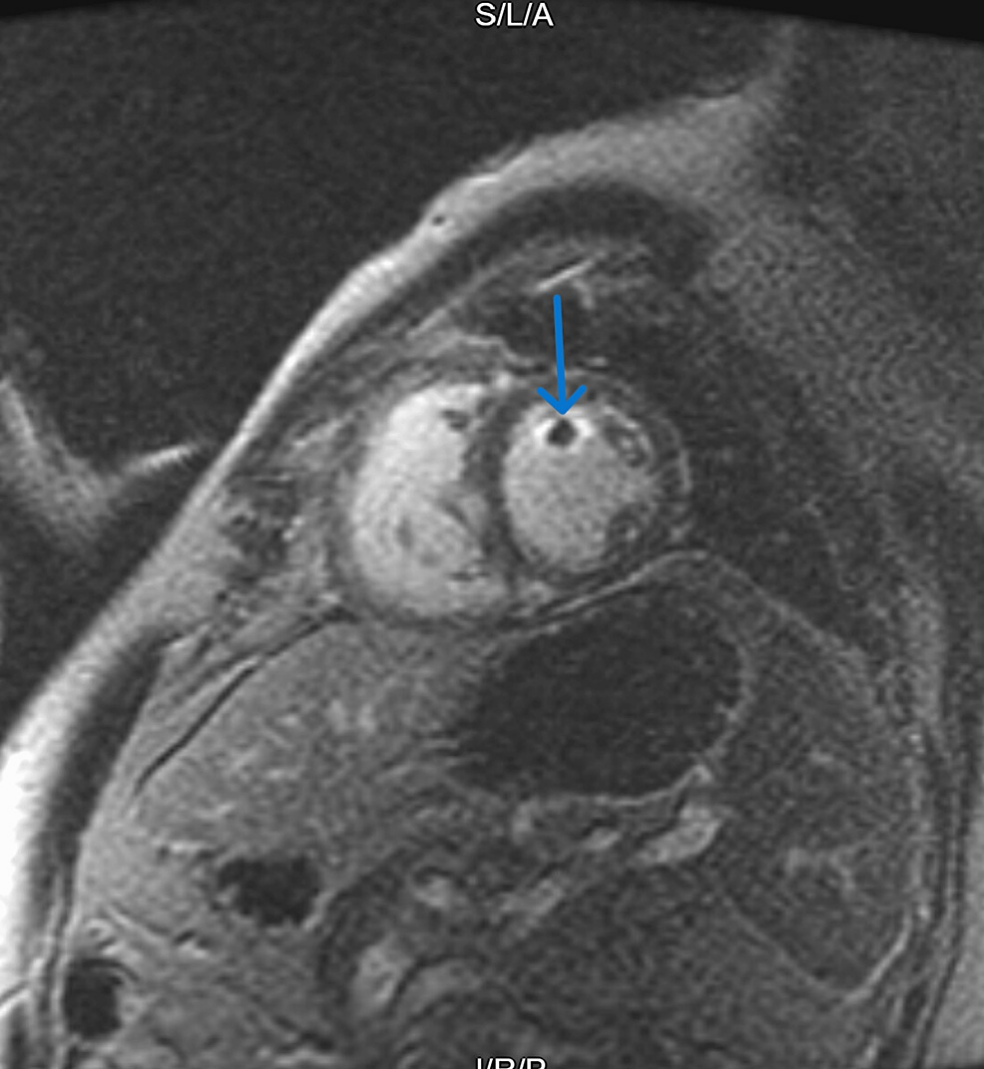
Cardiac MRI showing post-contrast peripheral enhancement of the left ventricular mass.

**Figure 3:**
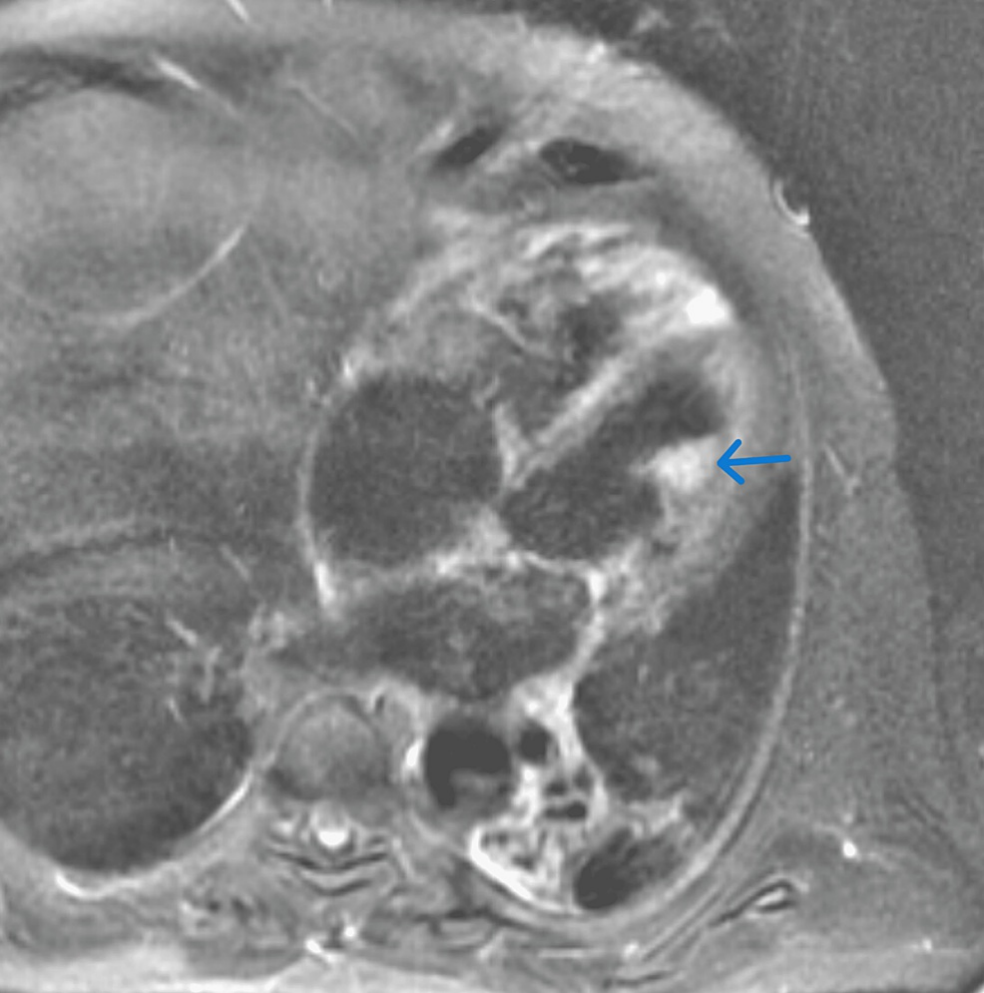
Cardiac MRI showing left ventricular hyperintense mass compared to surrounding myocardium.

Cardiac catheterization showed mild nonobstructive coronary disease. A concomitant right heart catheterization reported a mean RA pressure of 16 mmHg, mean PA pressure of 39 mmHg, PCWP of 29 mmHg, cardiac index of 1.65, and a transpulmonary gradient of 10. Subsequently, a cardiac MRI (CMR) demonstrated a 13 x 17 x 17 mm mobile mass with post-contrast peripheral enhancement arising from the anterior LV wall (Figures 1, 2, Video 1). This was felt to be probably hyperintense though limited due to motion artifact (Fig 3). CMR, PYP nuclear testing, and labs, including iron studies, pheochromocytoma screen, and monoclonal gammopathy screen, were unremarkable for infiltrative cardiac disease.

**Video 1:** Cardiac MRI showing mobile left ventricular mass attached to the anterior wall.

Due to severely compromised cardiac reserve, the patient was deemed high risk for surgical resection, and he also wanted to have more time to think about the surgical treatment. Thus, he was discharged home on goal-directed medical therapy with close heart failure clinic follow-up with potential evaluation for advanced heart failure therapies in the future. Unfortunately, he was lost to follow-up and was admitted three years later for a GI bleed. His most recent echocardiogram showed a 2.8 x 2.8 cm undulating echoic mass attached by a stalk to the anterolateral wall of the LV (Video 2). Fortunately, the patient had no embolic events, with only a slight increase in size. He will be followed up in the heart failure clinic.

**Video 2:** 2D echocardiogram showing 2.8 x 2.8 cm undulating echoic mass attached by a stalk to the anterolateral wall of the left ventricle.

## Discussion

Benign cardiac tumors may be detected incidentally, or they can present in multiple ways depending mainly on the location and sometimes on their type, for example, the constitutional symptoms frequently reported in cases of myxomas. In addition, clinical manifestations of malignant cardiac tumors also depend on size, friability, rate of growth, and invasiveness. Nevertheless, the most common symptoms are dyspnea, palpitations, syncope, systemic embolization, and arrhythmias.

Most PCT occur in atrial chambers or in association with valves. LV tumors are rare [3]. The most common LV intracavitary mass lesion is an LV thrombus which may be associated with underlying coronary artery disease or LV dysfunction due to ischemic or nonischemic cardiomyopathy [3,4]. TTE is the initial diagnostic modality to evaluate LV lesions. But CMR remains the gold standard to non-invasively characterize these lesions further. It is prudent to note here that a mass and thrombus are not mutually exclusive as, many times, the tumor acts as a nidus for thrombus formation. Mostly a thrombus is immobile, and CMR properties depend upon the chronicity of the thrombus. Cine-CMR characteristics of a thrombus include hypo- or iso-intensity to the surrounding myocardium and lack of enhancement. A chronic thrombus may show surface enhancement. In contrast, T1- and T2-weighted CMR depict the acute thrombus as hyperintense, while a chronic thrombus appears hypointense with these techniques [5]. A thrombus does not show early or late gadolinium enhancement due to a lack of vascularity [6].

The two most common benign PCT are myxoma and PFE. Myxoma is mostly located in the left atrium, followed by the right atrium. Only 3%-4% of myxomas occur in LV [7]. Myxoma is a mobile mass on cine-CMR and shows up as iso-intense or heterogenous on T1, hyperintense or heterogenous on T2-weighted images, and heterogenous on delayed images as well. The gold standard treatment for myxoma is surgical resection due to the high likelihood of systemic embolic and local cardiac complications, but it may not be possible in all cases [8].

PFE is the second most common PCT of endocardial origin. Its incidence has been increasing, likely related to the increasing diagnosis of antemortem cardiac masses due to the advent and widespread use of advanced diagnostic modalities [9]. PFE is usually a left-sided tumor (>95%) and accounts for more than three-quarters of all valvular tumors [10]. Prior radiation exposure, as well as HOCM, has a strong association with PFEs [9]. The aortic valve (aortic side) is the most involved location, followed by the mitral valve (left atrial side). PFE is found incidentally in most patients [2]. Because PFEs occur more frequently in high-pressure and high-flow chambers, the risk of systemic thromboembolism may even be higher when compared to myxomas (34% vs. 24%) [11]. Tumors are usually solitary, frond-like in appearance, and less than 1 cm in size. Diagnosis of PFE can be made with the help of echocardiography and CMR. Cardiac CT and transesophageal echocardiography may be helpful if the tumor location is atria or valvular, respectively. TTE may show a small, mobile mass with a short, thin stalk. It appears as hypointense on cine CMR images, isointense on T1, and isointense, hypointense, or hyperintense on T2-weighted imaging [5,12,13].

Curative surgery is recommended for all symptomatic PFEs. However, controversy exists in asymptomatic PFEs. Surgery is also recommended for asymptomatic, highly mobile, more than 1cm in size, and left-sided tumors [11]. Unfortunately, surgery might not be possible in all cases, similar to our patient. In these instances, there is no consensus on a medical treatment strategy for PFEs. In patients managed medically, there is up to a 6% risk of stroke at one year, which increases to 13% at five years, and it is significantly higher than matched population [14]. There is no data to support the use of long-term oral anticoagulation in these cases. Non-operative management also has higher mortality. More studies, follow-ups, and newer minimally invasive techniques are needed to benefit such patients who cannot undergo traditional surgical intervention for these lesions [14]. In our patient, the diagnosis of PFE may be favored over myxoma for several reasons: 1) due to prior radiation exposure, 2) very slow growth over 10 years, and 3) its location in the LV cavity, which would be less likely for a myxoma.

## Conclusions

Although CMR, with its tissue characterization properties, remains the diagnostic modality of choice to evaluate cardiac tumors, diagnosis, and subsequent appropriate therapeutic intervention remain challenging in some instances. Previous radiation is associated with cardiac PFE development, hinting toward its hamartomatous origins. Newer minimally invasive surgical techniques are needed to prevent complications and associated mortality and morbidity in select patients who are poor surgical candidates.

## References

[1] Poterucha TJ, Kochav J, O'Connor DS, Rosner GF (2019). Cardiac tumors: Clinical presentation, diagnosis, and management. Curr Treat Options Oncol.

[2] Bruce CJ (2011). Cardiac tumours: diagnosis and management. Heart.

[3] Viscuse PV, Bartlett DJ, Foley TA, Michelena HI (2018). Post-ischaemic exuberant left ventricular mass: thrombus vs. tumour-case report. Eur Heart J Case Rep.

[4] Dinesh Kumar US, Shetty SP, Sujay KR, Wali M (2016). Left ventricular mass: a tumor or a thrombus diagnostic dilemma. Ann Card Anaesth.

[5] Li X, Chen Y, Liu J (2020). Cardiac magnetic resonance imaging of primary cardiac tumors. Quant Imaging Med Surg.

[6] Hong YJ, Hur J, Kim YJ (2011). The usefulness of delayed contrast-enhanced cardiovascular magnetic resonance imaging in differentiating cardiac tumors from thrombi in stroke patients. Int J Cardiovasc Imaging.

[7] Reynen K (1995). Cardiac myxomas. N Engl J Med.

[8] Khan WJ, Asif M, Nadeem I (2023). Asymptomatic pulmonic valve mass: a diagnostic and therapeutic dilemma. Cureus.

[9] Elbardissi AW, Dearani JA, Daly RC, Mullany CJ, Orszulak TA, Puga FJ, Schaff HV (2008). Survival after resection of primary cardiac tumors: a 48-year experience. Circulation.

[10] Gowda RM, Khan IA, Nair CK (2003). Cardiac papillary fibroelastoma: a comprehensive analysis of 725 cases. Am Heart J.

[11] Mariscalco G, Bruno VD, Borsani P, Dominici C, Sala A (2010). Papillary fibroelastoma: insight to a primary cardiac valve tumor. J Card Surg.

[12] Mousavi N, Cheezum MK, Aghayev A (2019). Assessment of cardiac masses by cardiac magnetic resonance imaging: histological correlation and clinical outcomes. J Am Heart Assoc.

[13] Parwani P, Co M, Ramesh T (2020). Differentiation of cardiac masses by cardiac magnetic resonance imaging. Curr Cardiovasc Imaging Rep.

[14] Yong MS, Smail H, Saxena P (2016). Management of incidental papillary fibroelastoma: an update. Int J Cardiol.

